# Annotations

**Published:** 1894-03-24

**Authors:** 


### Annotations.

**Mr. Gladstone's Eyesight.**

All England is anxious to hear what hopes there may be of the perfect restoration of Mr. Gladstone's sight, and what prospect there is of an immediate or reasonably early recovery. It will surprise many to learn that less than a year ago his vision was not only remarkably acute, but was capable of standing a more prolonged strain than that of many men of less than half his age. It is not quite a year since *Hospitals and Asylums of the World* was published, and yet so recently as that Mr. Gladstone read through the whole of the four volumes—2,500 pages royal octavo—without being conscious of fatigue. This fact we have on the authority of the late Sir Andrew Clark. It is to be presumed, therefore, that if circumstances permit of a fairly early operation, the recovery of sight will be complete and perfect, and Mr. Gladstone may live to spend many years of learned and peaceful leisure in the full possession of that noble faculty of sight which throughout life has been the source of his greatest and most valued pleasures. Mr. Gladstone old and feeble is a pathetic but venerable object; Mr. Gladstone blind would be an object of poignant distress to his fellow-countrymen, though, we are consoled to think, not to himself. Moralising is out of date, and it has never been our forte; nevertheless, the most casual observer of the current of human affairs cannot but be struck with the contrast between last Easter and the present in so far as Mr. Gladstone and Sir Andrew Clark were concerned. Then Mr. Gladstone was at the height of his power and popularity and Sir Andrew Clark was at the zenith of his fame and success. Now, within one short year, Mr. Gladstone is nearly blind and Sir Andrew Clark lies dead in his last resting place. *Sic transit gloria mundi.*

**Dr. Louis Parkes—A New Surgical Censorship.**

It can hardly be doubted, even by those who may resent his opinions and interference, that Dr. Louis Parkes is animated by worthy motives and influenced by high public spirit in his present conflict with the authorities of the Chelsea Hospital for Women. Dr. Parkes has raised a most important question of public policy. Every outside person who may be interested in the subject ought, therefore, to lay aside all merely adventitious matter, such, for example, as charges against particular persons or institutions, and to deal separately and exclusively with the question of public policy raised. Narrowed down to a point, the issue is this—ought patients who die after surgical operations to be made the subjects of a coroner's inquiry, or rather, ought their cases to be laid before a "medical expert," such, for example, as the medical officer of health for the district, in order that he may decide whether or not a coroner's inquiry should be held? It is easy to see what this means: it means that an outside "medical expert," presumably the medical officer of health, is to sit in judgment upon the action of hospital surgeons when that action may have unfortunately been followed by death; it means, in short, an enormous curtailment of surgical liberty, and the investing of medical officers of health with new, wide, and certainly dangerous powers. We say a "curtailment of surgical liberty" advisedly, because no one supposes that deaths occurring after surgical operations in private practice would or could be placed under different regulations from those occurring in hospitals. The claim then of Dr. Louis Parkes is this, that every case of death after a surgical operation shall be submitted to an independent medical expert, the medical officer of health of course, and that he shall say whether or not a coroner's inquest is to be held. In the region of high transcendental theory this proposed departure may be right and desirable. But in actual practice it seems to us to be as uncalled for, as wrong-headed, and as injudicious a suggestion as could possibly have been made. Think of the tribunal before which hospital and private surgeons would have to appear in such an eventuality as is supposed! The medical officer of health would be their combined judge and jury. If medical officers of health as a class were all fit to be presidents of the College of Physicians we do not think such a thing as is contemplated ought to be done; but when the average medical officer of health is—well, is what he is—the thing seems to us, from a practical point of view, to be both ludicrous and intolerable.

**Chelsea and the Preventive Institute.**

The proposal to erect a building for the British Institute of Preventive Medicine at Chelsea appears to have aroused some local opposition. Certain anti-vivisectionists, taking occasion by this opposition, are worrying the promoters of the Institute on the one hand, and inflaming the feelings, without appealing to the reason, of the uninformed public on the other. Now this is not really the way to decide such a question. If the leaders of science are to be opposed in this matter, let them be opposed by dignified measures, represented by responsible persons, and carried out in an open, manly way. To set paid lecturers to worry at their heels, as an ill-conditioned dog snaps at a sober passer-by, is to inflict the greatest possible odium even upon anti-vivisectors themselves, and to retard the progress of science at the same time. No other result can spring from such conduct but the continuance of an undignified and ridiculous quarrel. The promoters of the Preventive Institute do not deserve this treatment. However their scheme be disapproved by some persons, it is certain that they are men of high character and of great scientific competence, and that they have in view the truly noble ends of increasing human knowledge and assuaging human ills. Such men may, of course, err as other men err; but at least their character and aims should protect them against futile and ridiculous worries and insults. If the anti-vivisectors think they have a good cause, let them make their appeal to the constituted authorities at Chelsea like fair and open-minded Englishmen. If they can convince the authorities, no doubt they may win their cause. If they cannot, let them surrender with dignity. The leaders of the anti-vivisectors owe it to themselves to do to men of science as they would be done by. If they carry out this principle they will promptly restrain their followers from the continuance of such tricky and un-English methods of procedure as were reported in Tuesday's *Times*.

